# Meta-analysis of the effects of monensin on growth and bloat of cattle on pasture

**DOI:** 10.1093/tas/txac031

**Published:** 2022-03-04

**Authors:** Shane Gadberry, David Lalman, Frank White, Sara Linneen, Paul Beck

**Affiliations:** 1 Department of Animal Science, University of Arkansas Division of Agriculture Cooperative Extension Service, Little Rock, AR 72204, USA; 2 Department of Animal and Food Sciences, Oklahoma State University Stillwater, OK 74048, USA; 3 Beef Business Unit, Elanco Animal Health, Greenfield, IN 46140, USA

**Keywords:** grazing, growing calves, meta-analysis, monensin, pasture

## Abstract

Monensin has been part of the beef production landscape for over 45 years. Although first approved for use in finishing cattle, it has since been approved for cattle in extensive production systems and has been an economical way to increase performance of forage-fed animals. This meta-analysis investigated the impacts of monensin on performance of stocker cattle on high-forage diets. The stocker performance analysis resulted from 38 experiments with 73 mean comparisons; bloat analysis was conducted with 12 experiments with 23 mean comparisons. The metaphor package (version 2.4-0) for R (version 4.0.3; www.r-project.org) was used to determine the overall effect size of monensin compared to a negative control. Each study’s *n*, means, and SEM or *P*-value was used to calculate the mean difference and estimate of within-study variance for responses of interest. Moderators of monensin response considered in the analysis were delivery method, dose, study duration, initial calf BW, diet ME and CP, and forage category. Initial BW and basal ADG averaged 236 ± 45.9 kg and 0.72 ± 0.28 kg, respectively. In the ADG analysis, the only significant moderator of those considered was length of the study (*P* < 0.01); as duration of the study increased, the ADG response to monensin decreased by 0.0007 kg/day. For the average 112-day length of study, the average monensin response was estimated to be 0.0784 kg/day increase in ADG, approximately 10% above controls. Sufficient information was presented in 18 citations representing 40 mean comparisons for determining the effect of monensin on BW at the end of the experiment. The response model (*P* < 0.01) for ending BW, kg = 22.3–0.05 (initial calf BW, kg). Thus, for the average initial BW of 235 kg the average monensin response was estimated to be 10.6 kg increase in average ending BW. The incidence (−20%) and severity (−0.7 bloat score) of bloat was found to be reduced in bloat-prone pastures. There is ample evidence that monensin increases performance of growing calves on high forage diets along with reducing the incidence and severity of bloat.

## INTRODUCTION

Since its approval for finishing cattle in 1975 (Goodrich et al., 1984), monensin has been used in both intensive and extensive beef production systems for over four decades. Monensin is a carboxylic polyether ionophore that selectively inhibits gram-positive bacteria, increasing propionate production and reducing the acetate:propionate ratio and methanogenesis, thereby improving ruminal metabolism energetic efficiency (Fuller and Johnson, 1981; Schelling, 1984). Schelling (1984) also noted reductions in bloat and risk of acidosis and improvements in ruminal nitrogen metabolism. Utilizing ionophores, such as monensin, are an economical way to increase ADG of forage-fed growing calves compared to nonmedicated supplements (Oliver, 1975; Potter et al., 1976; Boling et al., 1977; Males et al., 1979; Beck et al., 2016) or minerals (Fieser et al, 2007; Beck et al., 2014a). Traditionally, the FDA (2022) approved daily dose upper limit of 200 mg monensin per calf appeared to be the level of monensin most widely used in supplementation research, as it had consistently demonstrated improved BW gain of forage-fed cattle (Males et al., 1979; Rouquette et al., 1980; Potter et al., 1986). Nevertheless, a smaller number of studies suggest that lower levels of monensin may have performance benefits similar to 200 mg when provided to grazing cattle (Oliver, 1975; Boling et al., 1977). Monensin provided in free-choice mineral supplements has been proven to be effective in improving animal performance (Fieser et al., 2007; Beck et al., 2014a ); however, supplement consumption may be reduced and does not always provide the full recommended daily dose of 200 mg monensin/calf (Horn et al., 2005; Feiser et al., 2007; Beck et al., 2014a).

The 2015 survey of feedlot consulting nutritionist (Samuelson et al., 2016) showed that over 97% of their clients used ionophores in finishing diets. Adoption of growth promoting technologies by cow–calf producers is low at 14.1% of farms, but stocker operations were 1.49 times more likely to utilize these technologies than cow–calf producers (Pruitt et al., 2012). Producers are often unsure of the benefits of technologies, outcomes of technology use are often hard to measure in production settings, and often research results are inconclusive or contradictory which slows the adoption rates of technologies by producers. Use of growth promoting technologies on-farm are often difficult to measure in grazing cattle and thus the benefits are hard to enumerate. 

Meta-analysis is a statistical approach to combine responses among studies for establishing a generalized effect size. Meta-analysis methods can also compare effect size attributed to study level characteristics (called moderators) such as dose, duration of the study, or diet quality. Meta-analysis also aims to improve statistical power where original research succumbs to too much variation to establish statistical significance for the response mean difference. Thus, the objective of this paper is to present a meta-analysis of the responses of cattle in extensive stocker cattle production systems to dietary monensin addition for growth promotion and bloat reduction, to provide a basis for its utilization in these production systems, and to provide direction for avenues of future research.

## MATERIALS AND METHODS

As this report only used previously conducted research and published literature, no animals were used in this research and no animal care and use protocol was required.

### Stocker Research

#### Performance.

A literature search was conducted using databases from PUBMED, Google Scholar, Journal of Animal Science, Translational Animal Science, Applied Animal Science, Animal Production Science, and Oklahoma State University Animal Science Research Reports. Only data from experiments using diets and supplements that were similar between the negative controls and monensin treatments were used in this analysis. When multiple doses of monensin were compared to a control in a given experiment, only one dose was included in the analysis to avoid pseudo-replication of the control experimental units. This search generated 38 experiments with 72 means comparisons (Table 1). Monensin was supplied via hand-fed supplements either daily (34 mean comparisons) or on alternate days (9 mean comparisons), self-fed supplements (18 mean comparisons), or controlled ruminal release device (**CRRD**; 11 mean comparisons). Moderators of response to monensin considered in this analysis included: delivery method, dose, study duration, initial calf bodyweight (**BW**), diet ME (kcal/kg), diet CP (% DM basis), and forage type category. Forage type categories included as moderators in the analysis included perennial cool-season grasses (**CSP**; 8 mean comparisons), cool-season annuals (**CSA**, 21 mean comparisons), introduced warm-season grasses (**WSG**; 18 mean comparisons), native range (**NATIVE**; 10 mean comparisons), or dormant warm-season grasses (**DOR**; 6 mean comparisons). Just over 71% of the citations included analysis of the basal forage diet or adequate information to estimate the basal forage diet quality. The average forage quality was calculated to supply 16.9 ± 6.58% CP and 2.34 ± 0.32 kcal ME/kg. The basal forage diet ME ranged from 1.59 kcal ME/kg for bahiagrass a WSG in Florida to 2.95 kcal ME/kg for a CSA wheat pasture in Oklahoma. The basal diet CP ranged from 4.9% CP for a DOR tallgrass prairie site to 26.0% for a CSA wheat field in New Mexico. The initial BW in the 73 comparisons was 236 ± 45.9 kg with a basal ADG for controls that averaged 0.72 ± 0.284 kg, which ranged from 0.05 to 1.43 kg/day.

**Table 1.** Variable means, standard deviation, minimum and maximum for 38 publications with 73 experimental mean comparisons evaluating the effects of monensin supplementation on performance of growing cattle on high forage diets.

Variable	*N*	Mean	SD	Minimum	Maximum
Supplementation duration, d	73	112.6	50.8	51	370
Monensin dose, mg/d	73	150	57.4	23.2	300.0
Initial BW, kg	73	235.9	45.9	85.0	335.9
Basal ADG, kg	73	0.72	0.28	0.05	1.43
Forage ME, Mcal/kg	52	2.34	0.32	1.59	2.95
Forage CP, % DM basis	49	16.9	6.58	4.9	26.0

#### Bloat.

There were limited comparisons in the stocker cattle dataset (four experiments) with reported observations of bloat incidence and severity. Therefore, the search was expanded to include all published experiments reporting incidence or severity of bloat in cattle grazing bloat provocative pastures (alfalfa, perennial ryegrass, wheat, and combinations of cool-season annuals and legumes). This expanded search yielded 12 publications with 23 mean experimental comparisons (Table 2). Cattle used in these studies included cannulated steers (13 mean comparisons), dairy cows (6 mean comparisons), and growing stocker calves (4 mean comparisons). Eighteen mean comparisons included legume (alfalfa or clover) pastures and five mean comparisons included wheat or other small grain pastures. Of these publications, 12 reported the number of days monensin was provided, averaging 56 ± 46.7 days and ranging from 8 to 140 days; Monensin dose averaged 238 ± 120.6 mg monensin/day and ranged from 100 to 300 mg monensin/day.

**Table 2.** Variable means, standard deviation, minimum and maximum for 12 publications with 23 experimental mean comparisons evaluating the effects of monensin supplementation on incidence and severity of bloat in cattle on high forage diets.

Variable	*N*	Mean	SD	Minimum	Maximum
Supplementation duration, d	12	56	46.7	8	140
Monensin dose, mg/d	22	238	120.6	100	300.0

### Statistical Methods

The metaphor package (version 2.4-0) for R (version 4.0.3; www.r-project.org) was used to determine the overall effect size of monensin compared to a negative control (Viechtbauer, 2010). Each study’s n, means, and SEM or *P*-value was used to calculate the mean difference and estimate of within-study variance for responses of interest. The reciprocal of within-study variance was used for weighting each study’s contribution to the overall estimate of effect size and variability. A random-effects model was chosen to account for both within-study and between-study heterogeneity of variance. The model was fit using restricted-maximum likelihood and maximum-likelihood estimation. Maximum likelihood was used if restricted-maximum likelihood estimation did not converge. Maximum likelihood was also used when testing the effect of study characteristics on effect size estimate. Study characteristics evaluated with the stocker dataset included study duration (continuous), monensin dose (continuous), delivery method (categorical), forage type (categorical), and dietary energy supply (continuous). The influence of study characteristics on effect size was examined by comparing fit statistics between full (all characteristics included in the model) and reduced models (one and more study characteristics removed from the full model sequentially). Models were compared using analysis of variance with probability statistic calculated for the log-likelihood ratio. A final model was fit using restricted-maximum likelihood and either included study characteristics that influenced effect size or the model was reduced to a mean model (intercept only) when no study characteristics influenced effect size.

The effect size of continuous responses was modeled as mean difference with the exception of bloat score. Bloat score effect size included calculating effect size as a standardized mean difference (mean difference divided by the standard deviation) to account for studies reporting bloat score on different scales.

Analysis of bloat incident data presented its own challenges due to inconsistencies in how studies were analyzed and reported responses and whether studies analyzed these responses using individual animal or group as the experimental unit. Study counts were analyzed using the odds-ratio option of the metafor package. Count data were also re-calculated as proportions (*p*). The standard deviation for a proportion was calculated as the square root of *p*(1 − *p*). Bloat incidences from the stocker dataset were analyzed as proportions. Proportions were analyzed using the procedures described above for continuous responses.

Identified studies were inconsistent in reporting responses measured (e.g. ADG) and estimates of variability. For example, BW may have been reported as initial and final BW only, initial BW and ADG, or initial BW and BW gain. In some cases, standard error means were not reported but *P*-values or *P*-value thresholds were provided. In an effort to minimize study exclusion, missing continuous responses were calculated from available data (e.g. BW gain converted to ADG). For studies that did not report SEM for a continuous response variable or a response was calculated from available data, an estimate of SEM was determined arithmetically from *P*-values (Idris and Robertson, 2009; Gadberry et al., 2015). When *P*-value thresholds were provided, α = 0.05 was used for *P* < 0.05. When *P*-value was reported as *P* > 0.05, SEM was substituted based on comparison to other studies with similar *n* and mean difference for the response being analyzed. When neither *P*-value nor SEM were provided, first alternative was to use a substitute SEM based on other studies with similar *n* and mean difference, second alternative was to use the overall average SEM when the study had either similar n or mean difference as the other studies in the analysis. If a study could not meet any of these criteria for imputing a SEM, the study was excluded from the analysis. Imputing a SEM as described by the first alternative would help avoid excluding studies without a statistically significant response. Imputing a SEM as a second alternative would permit a study’s mean difference to contribute to the overall effect size, its weight toward the overall mean would only come from its contribution to between-study variability but not within-study variability as within-study variability would be equal to the overall average of within-study variances.

Similar to including study characteristics in the model, a dummy variable (“imputed”) was coded indicating which studies provided a SEM for the response (not imputed) and which studies had a calculated or substitute SEM (imputed). This variable was included in the model to determine whether or not including studies with imputed SEMs had a significant effect on the overall effect size estimate. The model was also fit without an intercept to observe the SEM of the imputed and nonimputed means. In general, including studies with calculated or substitute SEMs did not significantly affect the effect size of a response, but the SEM of imputed studies would contribute larger SEMs compared to the nonimputed studies. Allowing these studies to contribute to the overall effect increases the number of studies contributing to the overall effect and helps reduce bias toward studies that provided less response detail, especially if the within-study response was not statistically significant.

Boxplots and xy-plots were used to determine if any of the study characteristics would potentially skew effect size interpretation. Fitted models were assessed using provided fit statistics including test for residual heterogeneity, variance, test for study characteristics (i.e. test of moderators), and model results. The significance of model estimates was based on the normal distribution (*z*-value). Full and reduced models were compared as previously described. Standardized residual, funnel, and forest plots were examined to identify outliers. Using these tools, a study’s response would be removed from the analysis if there was sufficient evidence that the study’s mean difference, variance, or study’s characteristics were unique to all other studies and inclusion appeared detrimental to interpretation of the overall effect size.

## RESULTS

### Stocker Performance

#### Average daily gain.

Including studies with imputed SEM did not affect (*P* = 0.23) the model estimates of monensin on ADG of growing stocker calves; therefore, all studies with imputed SEM were included in the model. There were six experimental outliers removed from the analysis. Two experiments were removed from the model that included 300 mg/day monensin dose, two experiments were removed from the model that had initial starting BW < 125 kg, one experiment removed from the model that had monensin feeding days > 225, and one was removed from the analysis with a mean difference between control and monensin of −0.2 kg/day.

**Figure 1.**
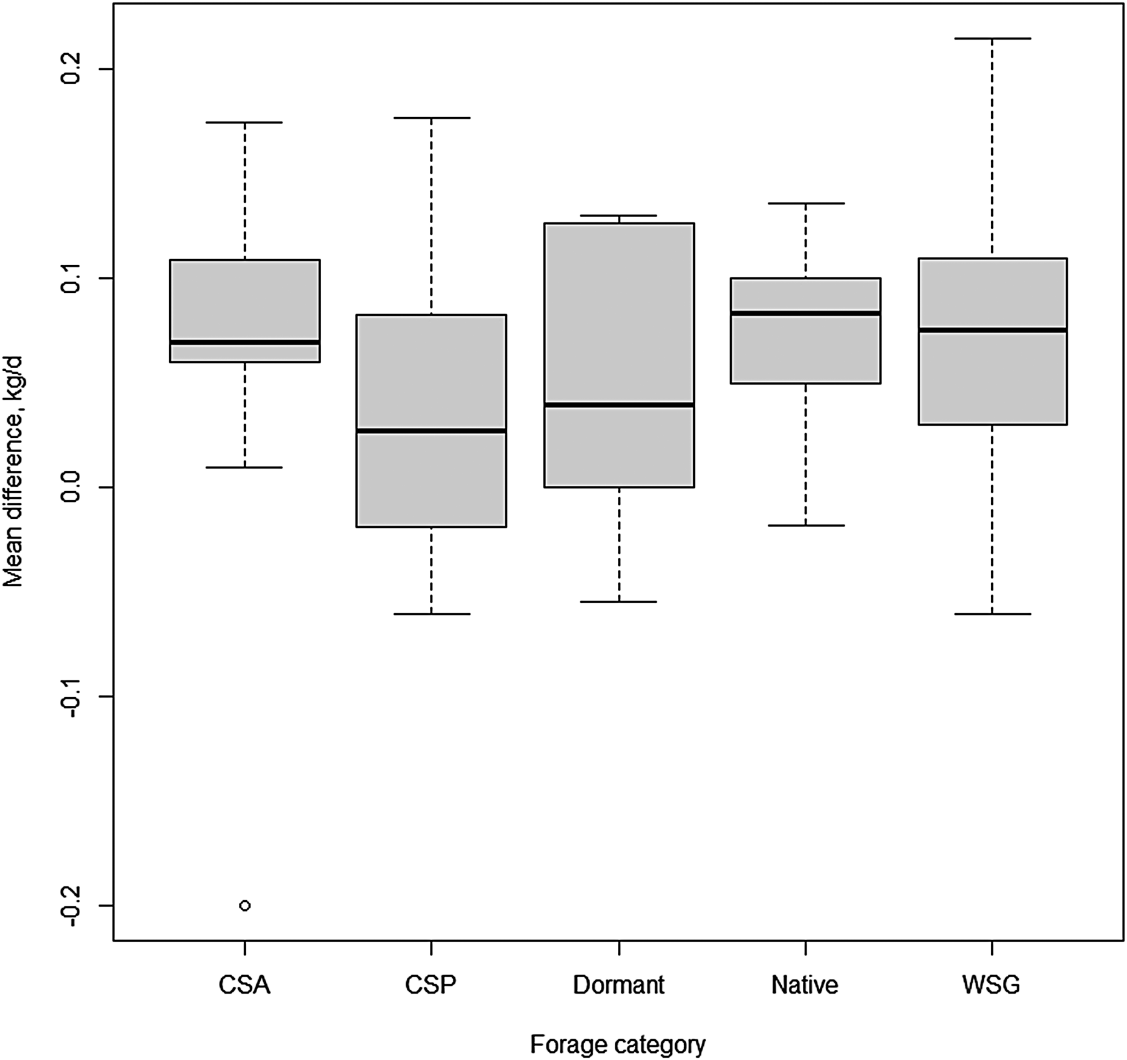
Boxplot of forage category as a modulator of ADG effect size for growing stocker calves receiving monensin. The five forage categories included: cool-season annuals (CSA), cool-season perennials (CSP), dormant warm-season grasses (DOR), native range (NATIVE), and introduced warm-season grasses (WSG).

**Figure 2.**
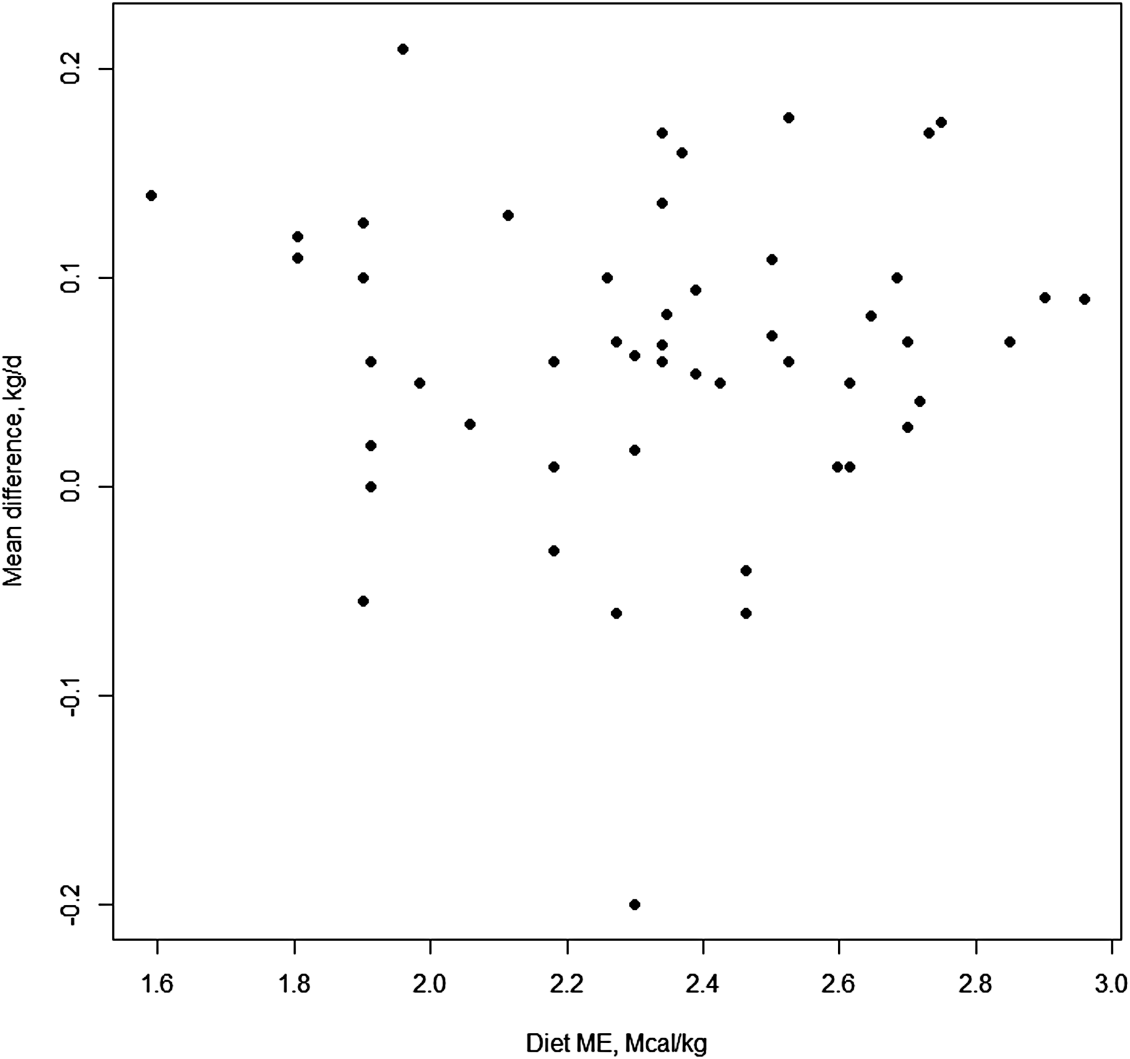
Xy-plot of basal diet ME (Mcal/kg) as a moderator of effect size for growing stocker calves receiving monensin.

There were 61 studies that included forage description adequate to determine forage category. The five forage categories included: cool-season annuals (CSA), cool-season perennials (CSP), dormant warm-season grasses (DOR), native range (NATIVE), and introduced warm-season grasses (WSG). The forage category response is presented in Fig. 1 to illustrate why forage category was not a significant moderator (*P* ≥ 0.22) to the ADG response by growing stocker calves receiving monensin. As with forage category, 52 experiments had sufficient data to estimate the dietary ME (Mcal/kg) of the basal forage diet and 49 had sufficient data to estimate dietary CP. Neither dietary CP nor diet ME (Fig. 2) were significant moderators (*P* ≥ 0.16) to the performance response by growing stocker calves to monensin.

**Figure 3.**
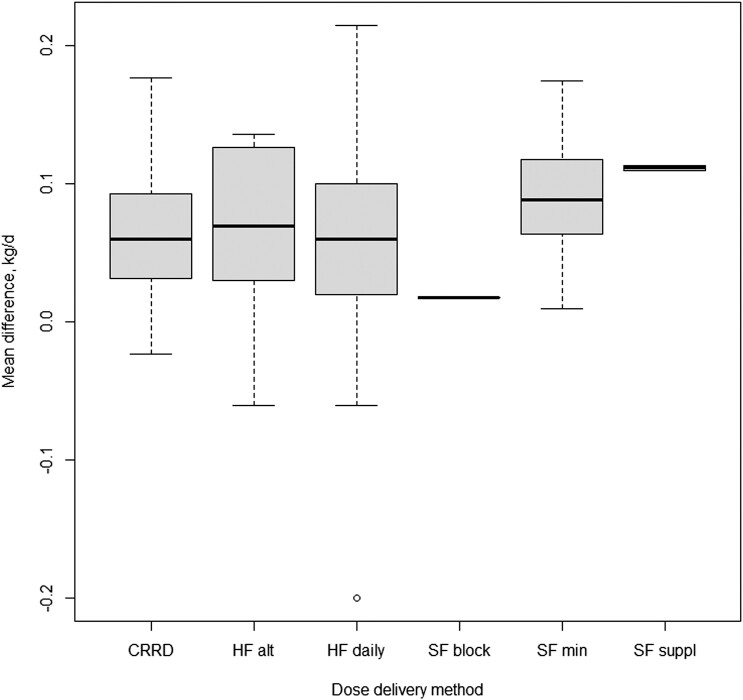
Boxplots for monensin delivery methods as a moderator to ADG effect size of growing stocker calves receiving monensin. The monensin delivery methods were categorized as: controlled ruminal release device (CRRD), hand-fed supplements supplied on alternate days (HF alt), hand-fed supplements supplied daily (HF daily), self-fed provided in a block (SF block), self-fed supplied in a complete mineral supplement (SF min), and self-fed supplements provided in the meal or pelleted form (SF suppl).

**Figure 4.**
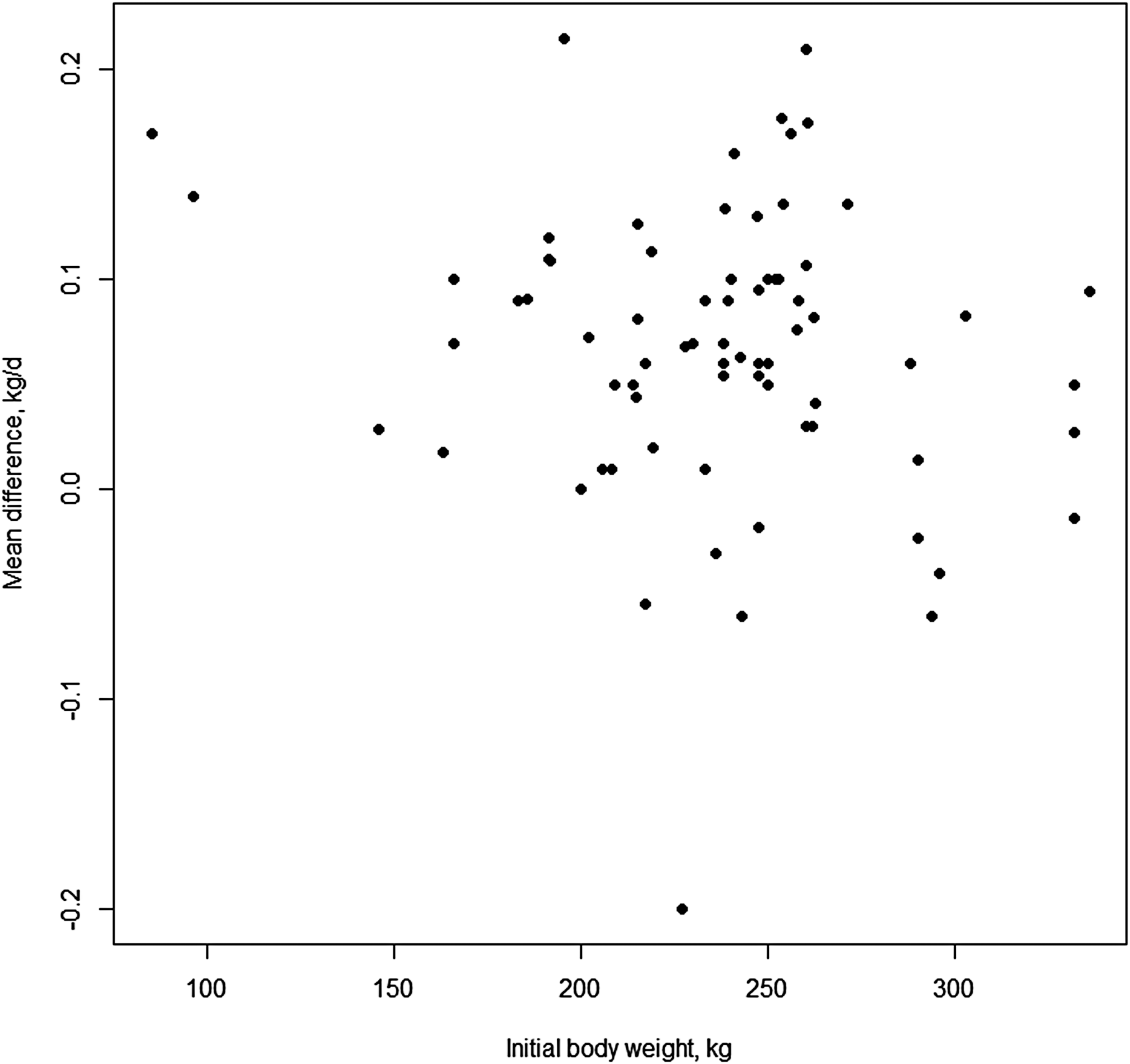
Xy-plot of initial BW of steers enrolled in the study as a moderator to the effect of monensin on ADG of growing stocker calves for all studies in the initial dataset.

**Figure 5.**
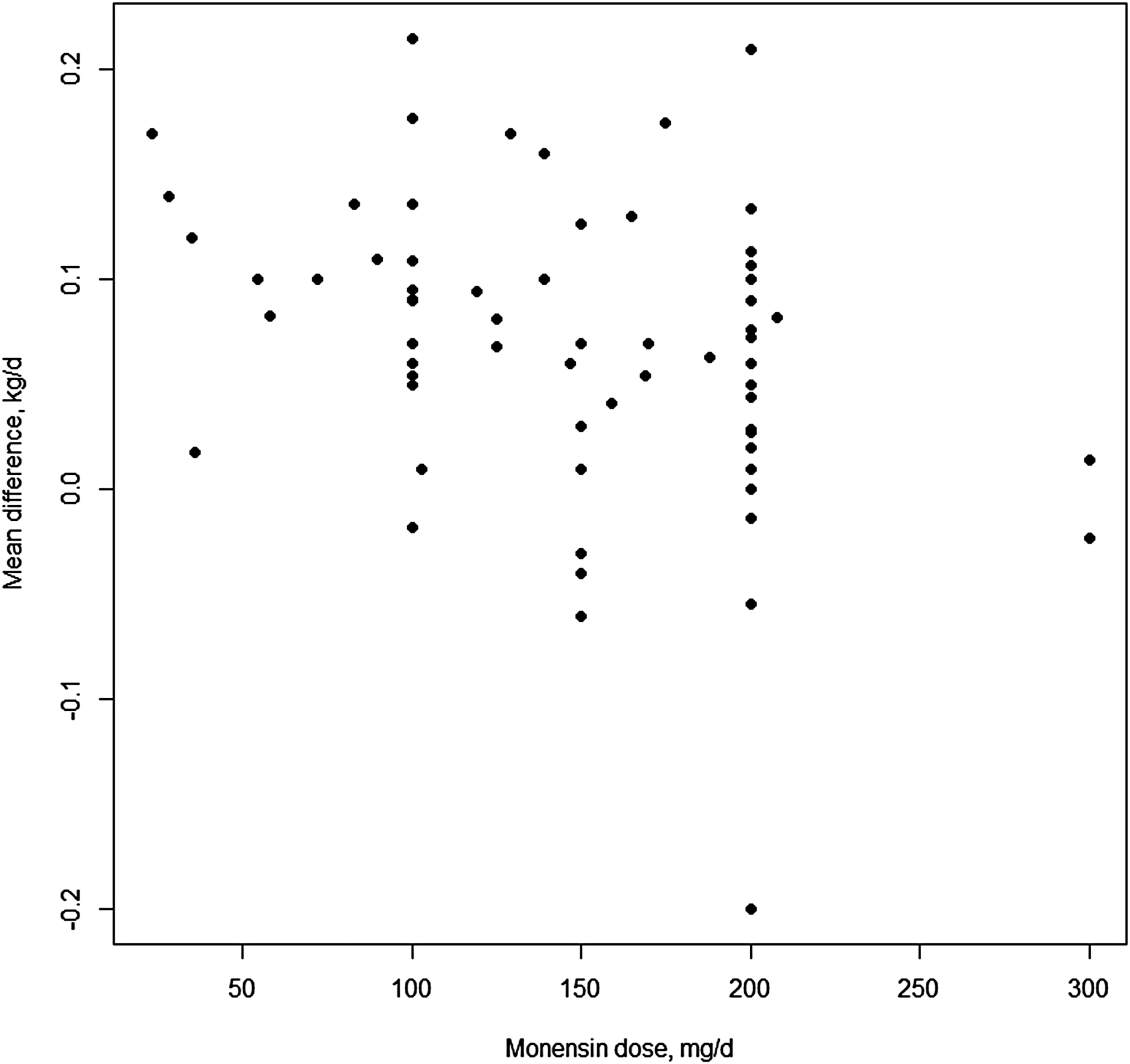
Xy-plot of monensin dose (mg/d) as a moderator of ADG of growing stocker calves receiving monensin for all studies in the initial dataset.

**Figure 6.**
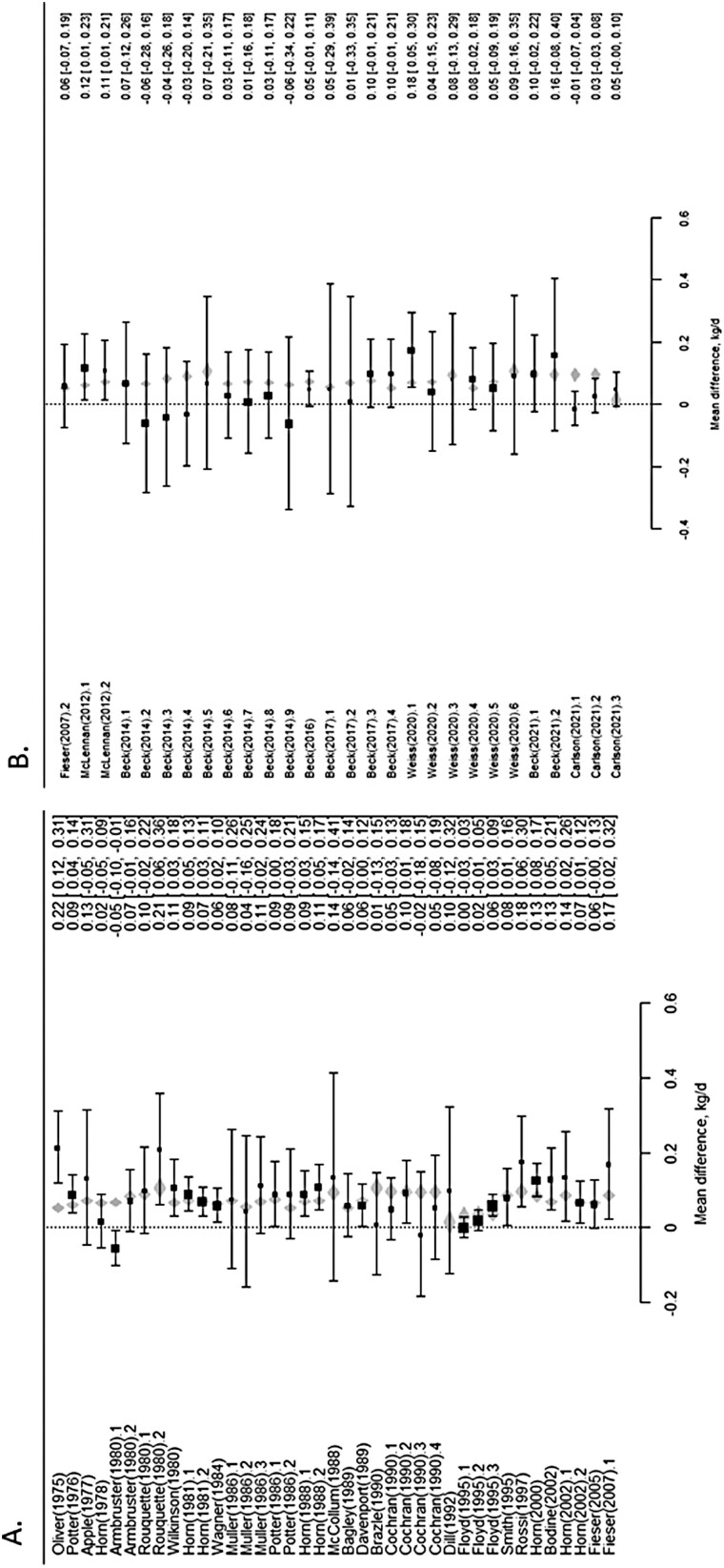
Forest plot of studies A) 1–39 and B) 40–78 for ADG mean difference of growing stocker calves receiving monensin.

**Figure 7.**
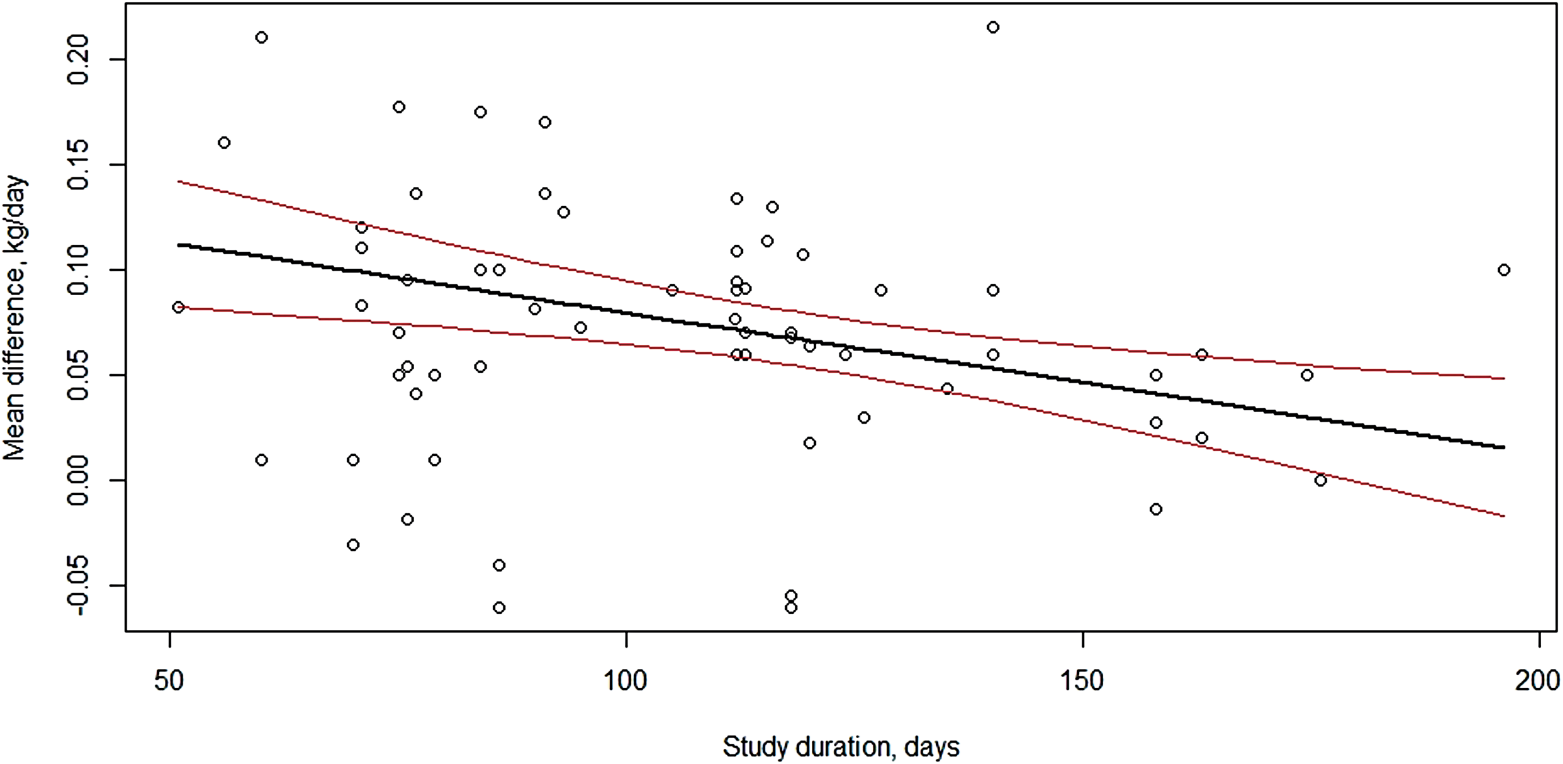
Xy-plot of study duration as a moderator or ADG of growing stocker calves receiving monensin. Study duration affected mean ADG response (*P* < 0.01) of growing stocker calves receiving monensin. The final model resulted in an overall mean response to monensin estimated by: Monensin response (increased BW kg/day) = 0.1459–0.0007 (duration of study, days).

The study moderators: delivery method (Fig. 3), initial BW of test animals (Fig. 4), dose (mg/day; Fig. 5) and length of experiment were from the full dataset and contained 67 mean comparisons following the removal of the six outlier experiments. The monensin delivery methods (Fig. 3) were categorized as: controlled ruminal release device (CRRD), hand-fed supplements supplied on alternate days (HF alt), hand-fed supplements supplied daily (HF daily), self-fed provided in a block (SF block), self-fed supplied in a complete mineral supplement (SF min), and self-fed supplements provided in the meal or pelleted form (SF suppl). Comparing models with and without delivery method indicated delivery method had no overall effect (*P* = 0.53) on ADG response. Likewise, including initial BW of calves enrolled in the experiments (Fig. 4) did not improve model fit (*P* = 0.67), neither did the inclusion of average monensin dose (*P* = 0.55) on the ADG response of growing stocker calves to monensin. The mean ADG difference of growing stocker calves receiving monensin for each study in the reduced dataset is presented in Fig. 6a and 6b. Contrary to the other study characteristics examined in this analysis, study duration was found to have a significant effect (*P* < 0.01) on ADG response of growing stocker calves to monensin (Fig. 7), such that for each day in study length ADG response was decreased by 0.0007 kg for each additional day in study duration.

The final model resulted in an overall mean response to monensin estimated by: Monensin response (increased BW kg/day) = 0.1459–0.0007 (duration of study, days). For the average 112-day length of study (Table 1), the average monensin response was estimated to be 0.0784 kg/day increase in ADG (Fig. 7).

#### Stocker calf BW.

**Figure 8.**
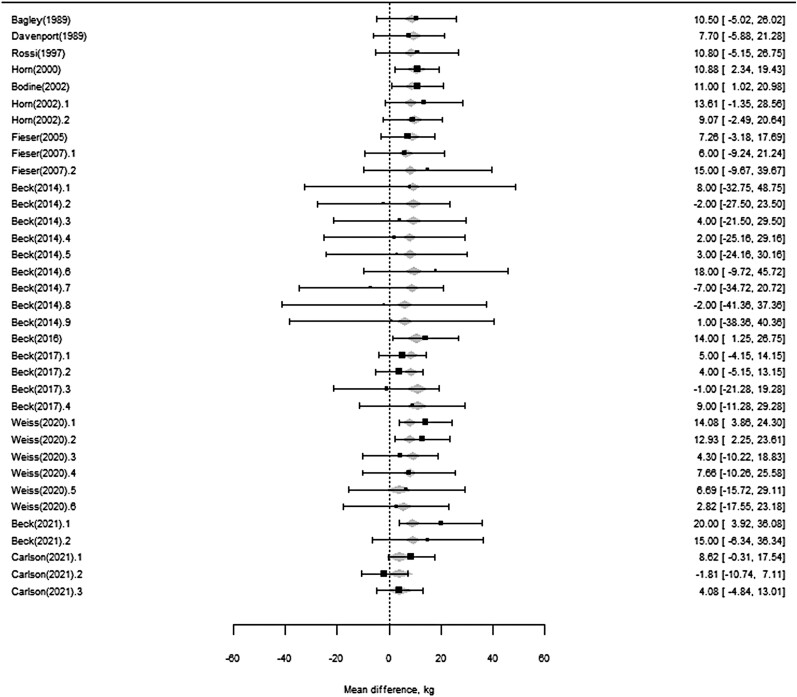
Forest plot of mean response of steer BW at the end of the experimental period due to monensin.

Sufficient information was presented in only 18 citations representing 40 mean comparisons for determining the effect of monensin on BW at the end of the experiment. A reduced dataset was utilized with outlier studies removed that had initial BW <150 kg and duration of study > 200 days. Length of study (*P* = 0.92), monensin dose (*P* = 0.58), or forage category (*P* = 0.43) did not improve model estimation of mean difference in BW at the end of the experiments due to monensin. Initial BW tended (*P* = 0.07) to decrease the mean response of ending BW to monensin by 0.05 kg for each kilogram increase in initial BW. The final model resulted in an overall mean response to monensin  (Fig. 8) estimated by: Monensin response (increased ending BW, kg) = 22.3–0.05 (initial calf BW, kg). Thus, for the average initial BW of 235 kg (Table 1) the average monensin response was estimated to be 10.6 kg increase in average ending BW (Fig. 8) with an average trial duration of 112 days.

### Bloat Response to Monensin

#### Severity of bloat.

**Figure 9.**
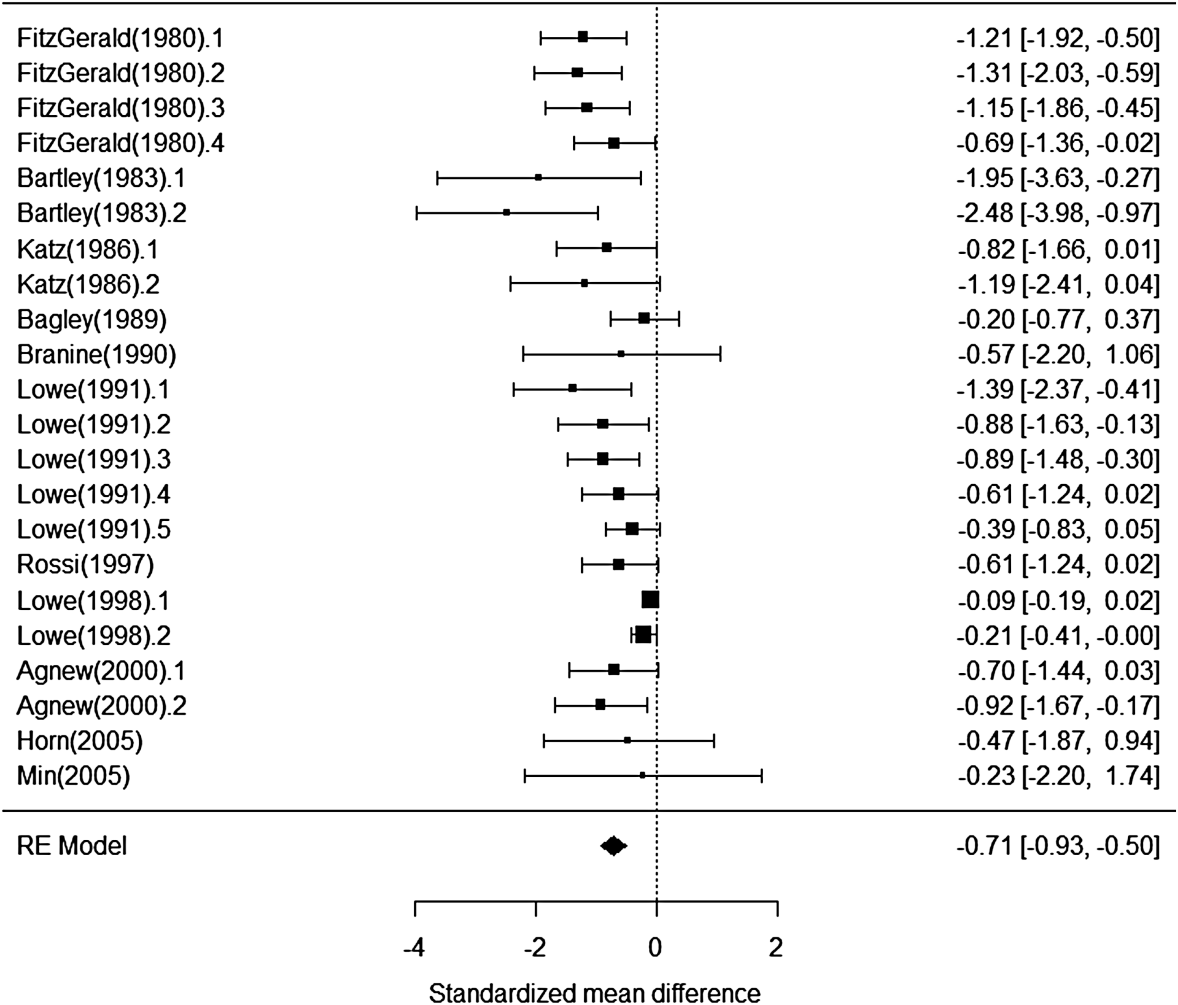
Influence of monensin on the standardized mean difference in bloat score for cattle grazing bloat provocative pastures.

Bloat scores were accessed in 22 mean comparisons, of which 16 did not report SEM. For these 16 responses, an estimated SEM was derived mathematically using the treatment *n*, mean, and *P*-values. A test for the effect of including studies with imputed SEM showed that including these in the analysis did not influence the estimates of monensin’s effect on bloat score (*P* = 0.43). Bloat scores originated from different scales, thus the effect size in this analysis was standardized as a standardized mean difference, which was the difference between means divided by the standard deviation. The analysis indicated that neither dose (*P* = 0.94) nor delivery method (*P* = 0.29) influenced differences in effect size among studies. The overall effect of monensin on bloat score was −0.71 ± 0.11 mean reduction in bloat score (Fig. 9). The average maximum possible score for the studies used in this analysis was 4.4. The average reported severity of bloat for controls was 1.3.

#### Incidence of bloat.

**Figure 10.**
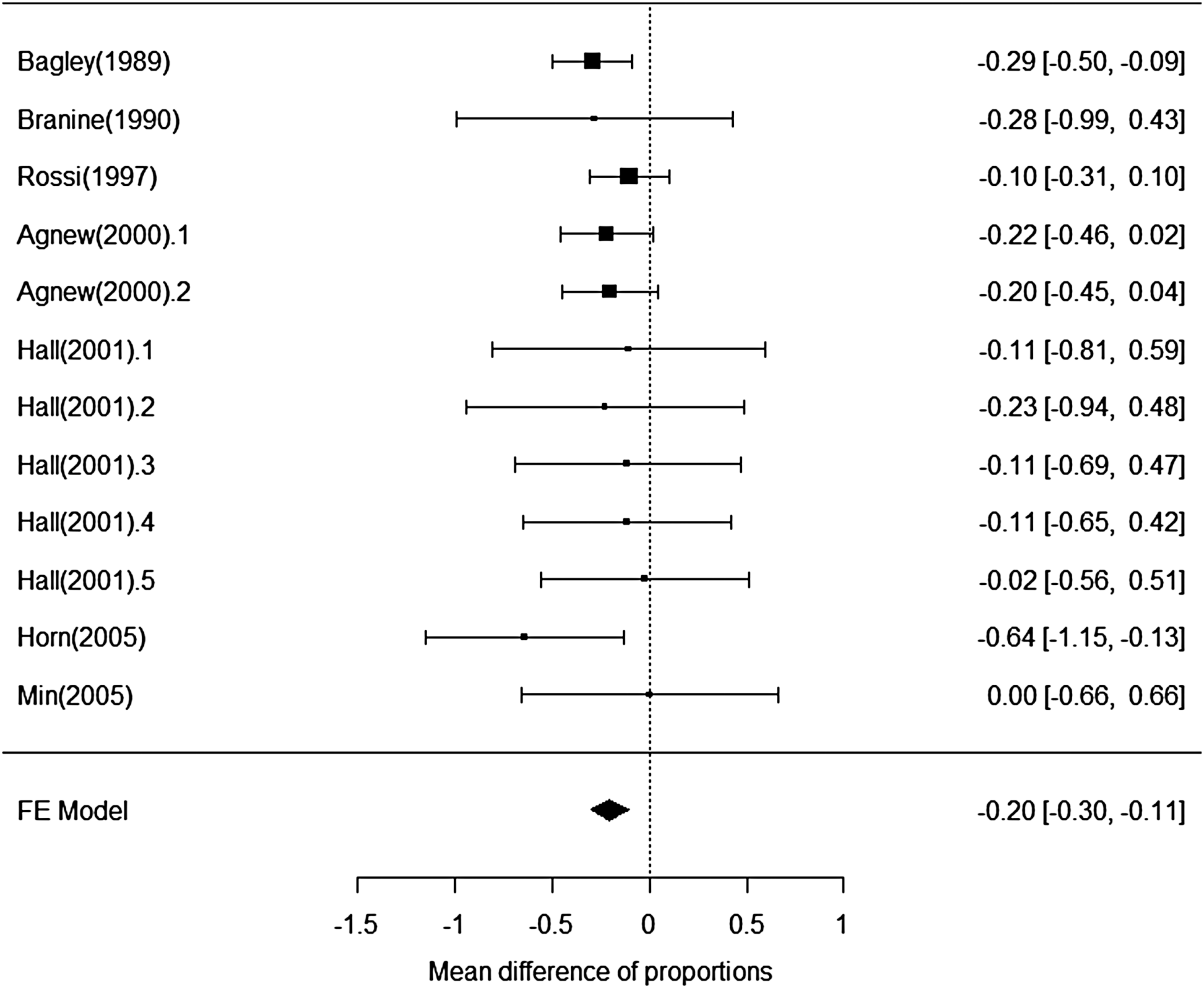
Influence of monensin on the mean difference in incidence of bloat in cattle grazing bloat provocative pastures.

The analysis of bloat incidence was based on 12 studies with either the reported or calculated proportion of bloat. The average bloat incidence reported in these studies was 28.5% for controls. Monensin doses were within a narrower range than that encountered in the analysis of performance of growing stocker calves (100–300 mg/day) and were delivered either in a hand-fed supplement or dosed directly into the rumen via either rumen cannula or CRRD (Table 2). The test of moderators to the effect of monensin on incidence of bloat indicate that the addition of dose (*P* = 0.98) or delivery method (*P* = 0.88) to the model did not improve model fit. Among studies, monensin decreased (*P* < 0.01) the incidence of bloat by 0.2 ± 0.05 or 20 percentage units (Fig. 10).

## DISCUSSION

### Study duration.

In the current analysis, duration of the study was found to be a significant moderator for ADG response to monensin (Fig. 5), such that for each day in study length ADG response was decreased by 0.0007 kg. The final model resulted in an overall mean response to monensin estimated by: Monensin response (increased BW kg/day) = 0.1459–0.0007 (duration of study, days). For the average 112-day length of study (Table 1), the average monensin response was estimated to be 0.0784 kg increase in ADG (Fig. 6), a 10.5% increase from the basal ADG of controls.

The stocker analysis was sufficient in scope to determine that study forage type and basal diet nutritive value was not influential on performance response to monensin by growing calves. The stocker calf analysis did determine that length of the grazing period had a negative influence on ADG response to monensin.

The significance of study duration as a moderator to gain response of growing stocker calves to monensin indicates there may be increasing tolerance of ruminal microbes to monensin. The lack of significant effects of forage type category and forage metabolizable energy and crude protein indicates that changes in forage quality during experiments with longer study duration was likely not the cause of the decline in monensin gain response observed, it may portend that there was ruminal tolerance of microbes over the course of longer studies. Duffield et al. (2012) indicated that the overall feed efficiency response to monensin in finishing diets was a 6.4% improvement but this has decreased to 2.5–3.5% improvement in the last two decades leading to discussion of reduced responses to monensin due to increasing tolerance to monensin over time. The reduction in feed efficiency gain response to monensin in feedlot settings (Duffield et al., 2012), also corresponds with changes in finishing diets and management over that same time (Landis, 2018). Ruminal bacteria previously exposed to monensin in vivo have exhibited noninheritable monensin tolerance in vitro that was reversible (Simjee et al., 2012). Weiss et al. (2020) conducted a series of experiments designed to determine if use of monensin during the stocker phase impacts the response to monensin during the finishing phase. In this study, there was no indication that feeding monensin during the stocker phase impacted gain or feed efficiency response to monensin fed during the finishing phase (stocker × finishing monensin feeding interaction, *P* ≥ 0.49). Steers fed monensin during finishing had reduced DMI (5%), numerically increased ADG (1.63%) and improved feed efficiency (6.9%), regardless of previous exposure to monensin during the stocker phase. Feeding monensin was found to decrease the rumen microbial alpha diversity Shannon Index during the stocker phase, but tended to increase alpha diversity for these cattle during the transition to finishing phase diets (Weiss et al., 2019), indicating that the response of microbial population to monensin was diet dependent.

An analysis of developing replacement heifers indicates the response to monensin (Moore et al., 2021) may differ from growing stocker calves in the present analysis. Although monensin improved ADG in replacement heifers (Moore et al., 2021), this response was substantially less than the stocker calf response (0.03 vs. 0.078 kg/day increase, respectively). There were inadequate number of studies using heifer or bull calves to include sex as a moderator in the analysis, because most studies used in the stocker analysis consisted of solely growing beef steers but this was not entirely the case for all studies. In a few cases (Beck et al., 2014b) when heifers and steers were used in grazing experiments there was no sex by monensin treatment interaction, showing that the response to monensin was not sex dependent.

### Forage quality and pasture species category.

The response of stocker cattle ADG was not influenced by forage quality (ME or CP of the basal forage diet) or forage species category. This analysis was conducted using experiments from the gamut of expected grazing conditions that would be expected in common stocker production systems ranging from low quality dormant native range to highly digestible legume and cool-season annual grasses.

In support of the results of the current meta-analysis, Weiss et al. (2020) conducted stocker experiments with monensin supplied in self-fed complete mineral supplements with a range of forages such as free-choice hay, bermudagrass pasture, and wheat pasture in six different blocks and found no treatment by basal diet interactions in the analysis of ADG response to monensin inclusion. When looking within the current dataset, differences in response to monensin among studies are observed across the gamut of forage nutritive quality. On very low quality dormant native range pastures (4.9% CP and 2.1 kcal ME/kg), Bodine et al. (2002) showed a large positive response (+0.13 kg/day or 81%) increase to monensin (Fig. 6a; 0.16 vs. 0.29 kg/day for controls vs. monensin, respectively). For higher quality pastures, Rossi et al. (1997) and Fieser et al. (2007) showed performance increased with monensin by 0.17 kg/d (22–30% increase for high quality alfalfa and wheat pastures, respectively (Fig. 6a).

Bretschneider et al. (2008) found that monensin increased ADG by 0.075 kg/day, which was very similar to the results of the current meta-analysis. Bretschneider et al. (2008) also found that ADG response to monensin decreased as basal ADG increased, which was not evident in our analysis of forage diet and diet nutrient density moderators. It was surmised by Bretschneider et al. (2008) that basal ADG were indicative of contrasting forage qualities and were influential on the direction and magnitude of response to antibiotic growth promoters. This lead Bretscheider et al. (2008) to propose that cattle grazing high quality pastures have reduced response to monensin because growing cattle on these forages are reaching the upper limit of their genetic potential for growth. The current meta-analysis did not observe forage-type category (Fig. 1) or forage quality (measured as metabolizable energy (Fig. 2) or crude protein concentrations) as significant modulators to the response of monensin, which does not support the conclusions drawn by Bretschneider et al. (2008).

### Monensin dose.

The FDA (2022) approved upper limit for daily monensin dose is 200 mg/calf for growing calves on pasture, with an approved range of 50–200 mg/day. The majority of the doses from experiments used in this analysis was in the 100–200 mg/day range (Fig. 5) which likely impacted the significance of monensin dose as a moderator to animal growth performance. Monensin inclusion in mineral supplements offered ad libitum has been shown to substantially reduce mineral intake (Fieser et al., 2007; Beck et al., 2014a; Beck et al., 2021), Weiss et al. (2020) found that mineral intake decreased linearly as monensin concentration increased resulting in monensin doses ranging from 54 to 208 mg/d. Monensin inclusion across doses improved growth performance by 9.7% (from 0.96 to 1.05 kg/d) compared to an unmedicated control within the doses observed in this experiment and regardless of basal forage quality. These results from Weiss et al. (2020) are very similar to the 10.5% increase in growth performance found in the current meta-analysis. Weiss et al. (2020) concluded that intake of a self-fed monensin-containing mineral can be decreased by at least 50%, without impacting performance response of growing steers to monensin on forage-based diets. Oliver (1975) found grazing steers supplemented with 0, 25, 50, 100, or 200 mg monensin/d had the greatest gain response at 100 mg/d followed by the 50 mg/d, suggesting a lower level may result in better growth performance compared to a higher level of monensin. This contrasts with Potter et al. (1976), who supplemented grazing calves with 0, 50, 100, 200, 300, and 400 mg monensin/d and found that the optimal gain response occurred for calves consuming 200 mg/d monensin. Based on the data from previous research and the current experiment, the effect of monensin level on BW gain can be variable. However, regardless of monensin dose, positive gain responses are likely for grazing calves supplemented with an appropriate amount of monensin compared to cattle that do not consume monensin across a wide range of forage types, forage nutritive quality, and basal performance levels.

### Bloat.

The bloat analysis included both growing and mature animals, but even with the limited number of studies consisting of growing cattle, the decreased incidence and severity of bloat on pasture in response to monensin appears to be consistent for all cattle grazing bloat provocative pastures. The average bloat incidence for control cattle was 28.5%, so the 20-percentage unit reduction is substantial.

The bloat scoring system used across experiments was not consistent, thus the average score for controls and monensin treated cattle needs to be compared to the average maximum possible score to account for differences in scales between studies. The average maximum possible score for the studies used in this analysis was 4.4. The average reported severity of bloat for controls was 1.3, which would be termed “slight distension” on the scales most commonly utilized. The 0.7 unit decrease in severity of bloat is also quite substantial for cattle grazing bloat provocative small grain or legume pastures.

Frothy bloat is a major cause of concern for cattle grazing wheat, other small grain pastures, and legume pasture (Merck Veterinary Manual, 1986) and the etiology of bloat is similar across small grain and legume pasture types. Incidences of bloat and death losses from wheat pasture bloat have considerable range and can strike suddenly and without warning. The etiology of pasture bloat depends on forage conditions, weather, stocking rates, and other management for legume pastures (Bartley and Bassette, 1961) and small grain pastures (Horn et al., 1976; Horn et al., 1977) alike. Frothy bloat is caused by the buildup of ruminal gasses that occurs when fermentation gas production is greater than gas expulsion via eructation (Merck Veterinary Manual, 1986). Pasture frothy bloat is usually related to the formation of stable foam from a viscous slime layer on the top of the rumen mat formed from soluble proteins and carbohydrates released from the forage during digestion as shown in legume bloat by Bartley and Bassette (1961). Gases released through fermentation percolate through the slime layer thereby forming stable foam that entraps ruminal gasses, which build up in the rumen. The chemical composition of bloat provocative forages changes depending upon environmental growing conditions, the stage of plant growth or maturity and fertility level (Beck et al. 2013); therefore, forage nutritive quality affects the likelihood that stable ruminal foam will be formed when wheat or other bloat provocative forages are grazed (Horn et al., 1976).

A common strategy for managing bloat is to provide a supplement containing monensin to calves throughout the grazing period and substitute poloxalene (a bloat therapeutic feed additive) for monensin during times of bloat outbreaks (Horn et al., 2005; Horn, 2006). With this approach, cattle are accustomed to going to a feeder when poloxalene feeding is needed, while the increased BW gain from the monensin improves the economics of the total supplementation program. Calves should be accustomed to feeders and supplementation during preconditioning prior to turn out onto bloat provocative pastures and maintain supplementation during grazing rather than waiting until a challenge arises to introduce a novel supplement or supplement delivery program. Economic benefits will be gained from the increased gains from feeding monensin and the reduced incidence and severity of bloat allows for timing to acquire alternative feed additives to control bloat. Bloat is a serious metabolic disorder that causes reduced performance of grazing cattle (Pitta et al., 2016) along with the 2–3% mortality rate commonly associated with bloat. Although forage type was not a significant moderator of stocker performance, there were considerable studies conducted on wheat pasture, legumes and other bloat provocative pastures. A likely reduction in subacute bloat by monensin in the studies conducted on small grain or legume pastures may have increased the gain response to monensin in the stocker cattle analysis for calves on these bloat provocative pastures.

## CONCLUSIONS

There is an ample evidence that monensin increases performance of growing calves on high forage diets. The current analysis also confirms that the incidence and severity of pasture bloat is reduced by providing monensin on bloat provocative pastures. Questions rise regarding the duration of feeding and carry-over effects of feeding monensin in one phase of production on the response to monensin in subsequent production phases. The theory of ruminal tolerance to monensin during the duration of feeding needs to be examined through longitudinal studies with repeated sampling throughout the lifecycle of the growing and finishing animal for microbiome analysis. Research is also necessary to determine the effects of feeding monensin in one stage of production and how it impacts the response to monensin in subsequent stages of production.

## Supplementary Material

txac031_suppl_Supplementary_Material
