# Supplementary material for: Meta-analysis of the effects of monensin on growth and bloat of cattle on pasture
Source: Transl Anim Sci. 2022 Mar 4;6(2):txac031. doi: 10.1093/tas/txac031 (PMC9030155; doi:10.1093/tas/txac031)
Supplement: txac031_suppl_Supplementary_Material [file txac031_suppl_supplementary_material.docx]

**Supplemental Material 1:**

**Systematic Review Reference List for Stocker Gain and Bodyweight**

Apple, K. L., and D. R. Gill. 1977. The effects of monensin on weight gains of steers and heifers grazing fescue pasture during the winter. OSU ANSI Res Rep. MP-101 pp. 4-7.

Armbruster, S. L., D. J. Fossler, G. E. Selk, L. J. Phillips, G. W. Horn, and D. R. Belcher. 1980. Monensin and implants for steers grazing native range. OSU ANSI Res Rep pp93-95.

Bagley, C. P., and J. I Feazel. 1989. Influence of a monensin ruminal bolus on the performance and bloat prevention of grazing steers. Nutr. Rep. Int. 40:707-716.

Beck, P. A., A. P. Foote, W. L. Galyen, T. W. Hess, D. S. Hubbell, M. S. Gadberry, E. B. Kegley, and M. D. Cravey. 2021. Effects of bambermycin or monensin offered in self-fed mineral supplements on performance of steer calves grazing small grain pasture. Appl. Anim. Sci. 37:670-680.

Beck, P., W. Galyen, D. Galloway, E. B. Kegley, R. Rorie, D. Hubbell, J. Tucker, T. Hess, M. Cravey, J. Hill, and C. Nichols. 2016. Effect of supplementation of developing replacement heifers with monensin or bambermycins on gain and pregnancy rates. Prof. Anim. Sci. 32:619-626.

Beck, P. A., T. Hess, D. Hubbell, G. D. Hufstedler, F. Fieser, and J. Caldwell. 2014. Additive effects of growth promoting technologies on performance of grazing steers and economics of the wheat pasture enterprise. J. Anim. Sci. 92:1219-1227.

Beck, P. B. Stewart, S. Gadberry, J. Tucker, D. Hubbell, J. Butterbaugh, T. Hess, K. Coffey, and B. Rudolph. 2014. Effect of daily or alternate-day distillers grains supplementation with or without monensin on performance of grazing calves. Prof Anim. Sci. 30:515-526.

Beck, P. A., M. S. Gadberry, C. B. Stewart, H. C. Gray, T. J. Wistuba, M. D. Cravey, and S. A. Gunter. 2017. Effects of a blended garlic and cinnamon essential oil extract with and without monensin sodium on the performance of grazing steers. Prof. Anim. Sci. 33:176-185.

Bodine, T. N., H. T. Purvis, II, G. W. Horn, and D. A. Cox. 2002. Response by Mexican crossbred stocker steers to implant type and monensin feeding status during winter grazing of dormant forages. OSU ANSI Res Rep. P-993 Available online: <file:///C:/Users/paubeck/Downloads/2002%20Bodine%20Research%20Report.pdf> accessed December 8, 2021

Brazle, F. K., G. Kuhl, and D. L. Harmon. 1990. The effect of antibiotics and ionophores on the gain of stocker cattle grazing native grass pastures. Prof. Anim. Sci. 6:19-23.

Burnham, D. L., S. T. Morris, R. W. Purchas, and S. N. McCutcheon. 1997. Effects of Compudose and Rumensin, alone or in combination, on the growth, and carcass and meat quality of steers finished on pasture. New Zealand J. Agric. Res. 40:231-238.

Carlson, Z. E., K. Butterfield, L. J. McPhillips, G. E. Erickson, M. E. Drewnoski, and J. C. MacDonald. 2021. Effects of monensin and protein type on performance of yearling steers grazing smooth bromegrass pastures. Nebraska Beef Cattle Report. 18-20.

Cochran, R. C., E. S. Vanzant, J. G. Riley, and C. E. Owensby. 1990. Influence of intraruminal monensin administration on performance and forage use in beef cattle grazing early-summer bluestem range. J. Prod. Agric. 3:88-92.

Daenicke, R., K. Rohr, and H. J. Oslage. 1982. Effect of monensin on rumen fermentation, performance and body composition of growing bulls. Live Prod. Sci. 8:479-488.

Davenport, R. W., M. L. Galyean, M. E. Branine, and M. E. Hubbert. 1989. Effects of a monensin ruminal delivery device on daily gain, forage intake, and ruminal fermentation of steers grazing irrigated winter wheat pasture. J. Anim. Sci. 67:2129-2139.

Dill, T. O., H. A. Turner, and D. W. Weber. 1992. Effects of Zeranol and monensin on reproductive performance of replacement heifers. Prof. Anim. Sci. 8:30-35.

Fieser, B. G., G. W. Horn, and J. R. Kountz. 2005. Effect of increasining levels of monensin in an energy supplement for cattle grazing winter wheat pasture. OSU ANSI Res Rep. available online: <file:///C:/Users/paubeck/Downloads/2005%20Fieser%20Research%20Report%20(1).pdf> Accessed December 8, 2021

Fieser, B. G., G. W. Horn, and J. T. Edwards. 2007. Effects of energy, mineral supplementation, or both, in combination with monensin on performance of steers grazing winter wheat pasture. J. Anim. Sci. 85:3470-3480.

Floyd, C. R., H. T. Purvis, K. S. Lusby, and R. P. Wetteman. 1995. Effects of monensin and 4-plex on growth and puberty of beef heifers. Prof. Anim. Sci. 11:95-99.

Horn, G. W., T. L. Mader, S. L. Armbruster, and R. R. Frahm. 1981. Effect of monensin on ruminal fermentation, forage intake, and weght gains of wheat pasture stocker cattle. J. Anim. Sci. 52:447-453.

Horn, G., C. Gibson, J. Kountz, and C. Lunsford. 2002. Two-year summary: effect of mineral supplementation with or without ionophores on growth performance of wheat pasture stocker cattle. Proc. Wheatland Stocker Conference. Enid, OK August 29, 2002.

Horn, G. W, S. L. Armbruster, M. L. Scott, and V. L. Stevens. 1978. Effect of monensin on forage intake and weight gains of wheat pasture stockers. OSU ANSI Res Rep. MP-102:11-15. Available online: <http://beefextension.okstate.edu/reports/pages-OLD/research78.html> accessed December 8, 2021

Horn, G. W., W. A. Phillips, D. Von Tunglin, G. J. Vogel, L. H. Carroll, and M. A. Worthington. 1988. Effect of a monensin ruminal delivery device on weight gains of growing steers on wheat pasture. OSU ANSI Res Rep MP-125:133-136. Available online: <http://beefextension.okstate.edu/reports/pages-OLD/research88.html> accessed December 8, 2021

MCCollum, F. T., D. R. Gill, and R. L. Ball. 1988. Steer gain response to monensin and chlortetracyline addition to summer protein supplements. OSU ANSI Res Rep. MP125: 137-138. Available online: <http://beefextension.okstate.edu/reports/pages-OLD/research88.html> accessed December 8, 2021

McLennan, S. R., M. J. Callaghan, A. J. Swain, and J. F. Kidd. 2012. Effect of monensin inclusion in supplements for cattle consuming low quality tropical forage. Anim. Prod. Sci. 52:624-629.

Muller, R. D., E. L. Potter, M. I. Wray, L. F. Richardson, and H. P. Grueter. 1986. Administration of monensin in a self-fed (salt limiting) dry supplements or on alternate-day feeding schedule. J. Anim. Sci. 62:593-600.

Oliveira, R. A, P. Moriel, J.M.B. Vendramini, H. M. Silva, M. Vedovatto, J.N.M. Neiva, F.R.C. Miotto, M. Miranda, and D. P. Silva. 2020. Supplemental monensin affects growth physiology and coccidiosis infestation of early-weaned beef calves consuming warm-season perennial or cool-season anuual grasses. Appl. Anim. Sci. 36:108-117.

Oliver, W. M. 1975. Effect of monensin on gains of steers grazed on Coastal bermudagrass. J. Anim. Sci. 41:999-1001.

Potter, E. L., C. O. Cooley, L. F. Richardson, A. P. Raun, and R. P. Rathmacher. 1976. Effect of monensin on performance of cattle fed forage. J. Anim. Sci 43:665-669.

Potter, E. L., R. D. Muller, M. I. Wray, L. H. Carroll, and R. M. Meyer. 1986. Effect of monensin on the performance of cattle on pasture or fed harvested forages in confinement. J. Anim. Sci. 62:583-592.

Rossi, D. M., F. Navarro, and C. D. Grivel. 1997. Effect of monensin on weight gain and prevention of bloat in steers on an alfalfa meadow. Arch. Med. Vet. 29:279-282 dx.doi.org/10.4067/S0301-732X997000200013

Rouquette, F. M., Jr., J. L. Griffin, R. D. Randel, and L. H. Carroll. 1980. Effect of monensin on gain and forage utilization by calves grazing bermudagrass. J. Anim. Sci. 51:521-525.

Smith, S. C., J. D. Enis, and D. R. Gill. 1995. Effect of bambermycin on weight gain of summer stocker cattle. OSU ANSI Res Rep P-943:142-144. Available online: <http://beefextension.okstate.edu/reports/1995> accessed December 8, 2021

Wagner, J. F., H. Brown, N. W. Bradley, W., Dinusson, W. Dunn, N. Elliston, J. Miyat, D. Mowrey, J. Moreman, L. c. Pendlum, C. Parrott, L. Richardson, I. Rush, and H. Woody. 1984. Effect of monensin, estradiol controlled release implants and supplement on performance in grazing steers. J. Anim. Sci. 58:1062-1067.

Weiss, C. P., P. A. Beck, M. S. Gadberry, T. Hess, J. Hill, and D. Hubbell. 2020. Effect of monensin dose from a self-fed mineral supplement on performance of growing steers on forage-based diets. Appl. Anim. Sci. 36:515-523.

Wilkinson, J.I.D., W.G.C. Appleby, C.J. Shaw, G. Lebas, and R. Pflug. The use of monensin in European pasture cattle. Anim. Prod. 31. 159-162.

**Supplemental Material 2:**

**Systematic Review Reference List for Incidence and Severity of Pasture Bloat**

Agnew, K.E.M. C. A. Morris, and N. G. Cullen. 2000. Evaluation of a liquid formulation of monensin to control bloat in pasture-fed milking cows. New Zeal. Vet. J. 48:74-77.

Bagley, C. P., and J. I Feazel. 1989. Influence of a monensin ruminal bolus on the performance and bloat prevention of grazing steers. Nutr. Rep. Int. 40:707-716.

Bartley, E. E., T. G. Nagaraga, E. S. Pressman, A. A. Dayton, M. P. Katz, and L. R. Fina. 1983. Effects of lasalocid or monensin on legume or grain (Feedlot) bloat. J. Anim. Sci. 56:1400-1406.

Branine, M. E., and M. L. Galyean. 1990. Influence of grain and monensin supplementation on ruminal fermentation, intake, digesta kinetics and incidence and severity of frothy bloat in steers grazing winter wheat pasture. J. Anim. Sci. 68:1139-1150.

FDA. 2022. U. S. Food & Drug Administration Code for Federal Regulations Title 21. Available online: <https://www.accessdata.fda.gov/scripts/cdrh/cfdocs/cfcfr/CFRSearch.cfm?fr=558.355> Accessed February 22, 2022.

FitzGerald, R. D., E. C. Wolfe, R. H. Laby, and D. G. Hall. 1980. Beef production from lucerne and subterranean clover pastures 2. bloat occurance and effect of anti-bloat capsules. Aust. J. Exp. Anim. Husb. 20:688-694.

Hall, J. W., W. Majak, T. A. McAllister, and J. K. Merrill. 2001. Efficacy of Rumensin controlled release capsule for the control of alfalfa bloat in cattle. Can. J. Anim Sci. 81:281-283.

Horn, G. W., P. A. Beck, J. G. Andrae, and S. I. Paisley. 2005. Designing supplements for stocker cattle grazing wheat pasture. J. Anim. Sci. 83(E-Suppl.):E69-E78.

Katz, M. P. T. G. Nagaraja, and L. R. Fina. 1986. Ruminal changes in monensin- and lasalocid-fed cattle grazing bloat-provacative alfalfa pasture. J. Anim. Sci. 63:1246-1257.

Lowe, L. B., G. J. Ball, V. R. Carruthers, R. c. dobos, G. A. Lynch, P. J. Moate, P. R. Poole, and S. C. Valentine. 1991. Monensin controlled-release intraruminal capsule for control of bloat in pastured dairy cows. Australian Vet. J. 68:17-20.

Lowe, L. B. 1998. Prevention of bloat in pastured cattle - using monensin sodium controlled release capsule (CRC). Bovine Pract. 32:27-30.

Min, B. R., W. E. Pinchak, J. D. Fulford, R. Puchala. 2005. Effect of feed additives on in vitro and in vivo rumen characteristics and frothy bloat dynamics in steers grazing wheat pasture. Anim. Feed Sci Tech. 123-124:615-629.

Rossi, D. M., F. Navarro, and C. D. Grivel. 1997. Effect of monensin on weight gain and prevention of bloat in steers on an alfalfa meadow. Arch. Med. Vet. 29:279-282. dx.doi.org/10.4067/S0301-732X997000200013
